# Phenotypic and Functional Consequences of PLT Binding to Monocytes and Its Association with Clinical Features in SLE

**DOI:** 10.3390/ijms22094719

**Published:** 2021-04-29

**Authors:** Anaís Mariscal, Carlos Zamora, Berta Magallares, Tarek Carlos Salman-Monte, Mª Àngels Ortiz, Cesar Díaz-Torné, Iván Castellví, Héctor Corominas, Silvia Vidal

**Affiliations:** 1 Immunology Department, Hospital de la Santa Creu I Sant Pau, Biomedical Research Institute Sant Pau (IIB Sant Pau), 08041 Barcelona, Spain; amariscal@santpau.cat; 2 Laboratory of Inflammatory Diseases, Hospital de la Santa Creu I Sant Pau, Biomedical Research Institute Sant Pau (IIB Sant Pau), 08041 Barcelona, Spain; czamora@santpau.cat (C.Z.); mortiz@santpau.cat (M.À.O.); 3 Rheumatology Department, Hospital de la Santa Creu I Sant Pau, 08041 Barcelona, Spain; bmagallares@santpau.cat (B.M.); cdiazt@santpau.cat (C.D.-T.); icastellvi@santpau.cat (I.C.); hcorominas@santpau.cat (H.C.); 4 Rheumatology Department, Parc de Salut Mar/Hospital del Mar-IMIM, 08003 Barcelona, Spain; tareto4@gmail.com

**Keywords:** platelets, monocytes, lupus, immune modulation

## Abstract

Platelets (PLTs) can modulate the immune system through the release of soluble mediators or through interaction with immune cells. Monocytes are the main immune cells that bind with PLTs, and this interaction is increased in several inflammatory and autoimmune conditions, including systemic lupus erythematosus (SLE). Our aim was to characterize the phenotypic and functional consequences of PLT binding to monocytes in healthy donors (HD) and in SLE and to relate it to the pathogenesis of SLE. We analyzed the phenotypic and functional features of monocytes with non-activated and activated bound PLTs by flow cytometry. We observed that monocytes with bound PLTs and especially those with activated PLTs have an up-regulated HLA-DR, CD86, CD54, CD16 and CD64 expression. Monocytes with bound PLTs also have an increased capacity for phagocytosis, though not for efferocytosis. In addition, monocytes with bound PLTs have increased IL-10, but not TNF-α, secretion. The altered phenotypic and functional features are comparable in SLE and HD monocytes and in bound PLTs. However, the percentages of monocytes with bound PLTs are significantly higher in SLE patients and are associated with undetectable levels of anti-dsDNA antibodies and hematuria, and with normal C3 and albumin/creatinine levels. Our results suggest that PLTs have a modulatory influence on monocytes and that this effect may be highlighted by an increased binding of PLTs to monocytes in autoimmune conditions.

## 1. Introduction

Platelets (PLTs) have recently been recognized as immunoregulatory elements [1,2] that can modulate immune system responses by releasing soluble mediators (TGF-β, IL-1β, PF4, RANTES or MIP1α) or through interaction with immune cells [1,3,4,5,6]. In healthy individuals, PLTs preferentially bind to monocytes [7,8,9,10], mainly through P-selectin (CD62P)-PSGL-1 [11]. One consequence of this interaction is the promotion of cellular extravasation [12] via the up-regulation of expression and the functionality of integrins [13]. Another consequence is the induction of the expression and secretion of monocyte chemotactic protein-1 (MCP-1) and IL-8 [14]. Monocyte–PLT interaction decreases monocyte apoptosis [15] and leads to a phenotypic change of CD14+CD16- towards CD14+CD16+ monocytes [16] and to monocyte differentiation into macrophages [17]. However, it has recently been described that PLTs can also dampen inflammatory responses by increasing IL-10 and decreasing IL-6 and TNF-α release by monocytes [18,19]. These opposed effects of PLTs on innate immune cells may be ruled by the PLT activation state [20,21] or by the blood flow microenvironment [22].

It is well known that monocytes and PLTs play a crucial role in SLE pathology. On the one hand, a decreased number of circulating monocytes and an increased ratio of non-classical vs. classical monocytes were observed in SLE patients [23,24]. SLE monocytes also have an altered phenotype and function: an increased expression of adhesion molecules [25], Fc receptors for IgG and co-stimulatory proteins, and a dysregulated cytokine secretion (decreased production of IL-1 [26] but an increased production of TNF-α [27], IL-6 [28] and IL-10 [12,13]). The phagocytosis of apoptotic cell material is also impaired in monocytes from SLE patients [29] and correlates negatively with the activity index and anti-dsDNA antibodies. The failed monocyte/macrophage clearance of apoptotic cells leads to secondary necrosis and the release of novel autoantigen clusters to trigger and/or sustain autoimmunity in SLE [30]. On the other hand, SLE PLTs are involved in the FcγRIIA-mediated clearance of IgG immune complexes [31]. In fact, PLTs express CD40L, which in SLE patients triggers the production of type I interferon, promoting the production of autoantibodies by plasma cells [32].

In addition to CD62P-PSGL-1, there are other ligands that can be involved in the binding of PLTs to monocytes: GPIb-CD11b [33], CD40-CD40L [34], GPIIb/IIIa-CD11/CD18 [35], EMMPRIN (CD147/basigin)-CD147/GPIV [36,37], TREM-1-TREM-1 ligand [38] and PADGEM [39]. Most of these molecules are expressed on the surface of PLTs when they activate, and increased leukocyte-PLT complexes therefore suggest PLT activation [40]. However, each pair of molecules triggers different events in monocytes: an inflammatory function for CD62P-PSGL-1 and EMMPRIN (CD147/basigin)-CD147/GPIV ligation [36,37,41], while the triggering of anti-inflammatory functions is carried out through CD40L-CD40 [18].

Circulating monocytes with bound PLTs have been seen in healthy donors (HD) with unknown functional consequences. One possible consequence of the binding of PLTs to cells is to collaborate with monocytes/macrophages in eradicating bacterial infections. This mechanism has been proposed by Wong et al. as a transient “touch-and-go” interaction of PLTs with Kupffer cells [42]. In addition, there is an increased percentage of monocytes with bound PLTs in several inflammatory and autoimmune conditions, including atherosclerosis [43], type 1 diabetes [44], rheumatoid arthritis [37] and psoriatic arthritis [45]. Increased percentages of monocyte-PLT complexes in SLE have also been observed [8]. With the objective of determining the consequences of PLT binding to monocytes, we firstly studied the phenotypic and functional characteristics of monocytes with and without bound PLTs or activated PLTs in HD. Secondly, we compared the phenotypic and functional characteristics of monocytes with bound PLTs in HD and SLE patients, and finally, we associated monocytes with bound PLTs with the clinical and laboratory features of SLE patients. We found that monocytes with bound PLTs have altered phenotype and functions in both HD and SLE patients. SLE patients have higher percentages of monocytes with bound PLTs, and these are related to clinical features.

## 2. Results

### 2.1. Comparison of Phenotype and Function of Monocytes with and without Bound PLTs in HD

**Figure 1:**
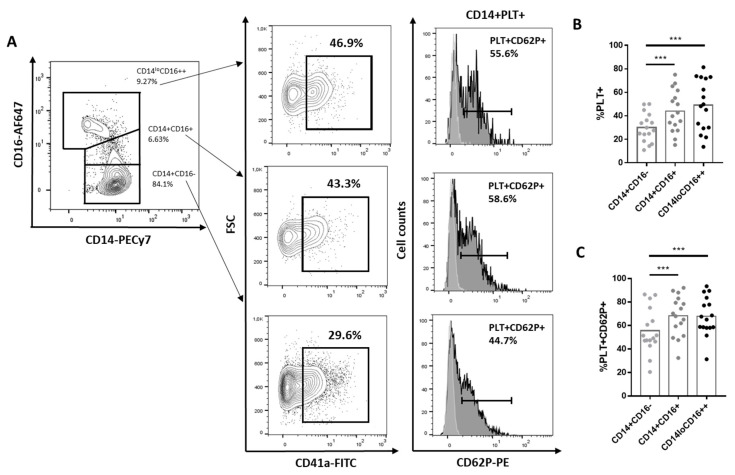
Percentage of CD14+CD16-, CD14+CD16+ and CD14+CD16++ with bound platelets. (**A**) A representative experiment showing the gating strategy for identifying monocyte subsets CD14+CD16-, CD14+CD16+ and CD14+ CD16++. CD14+ with bound PLTs (CD14+CD41a+) and monocytes with activated bound platelets (CD14+CD41a+CD62P+) (dark histograms). Light grey histograms represent CD62P expression on each subpopulation of monocytes without PLTs. (**B**). Percentage of CD14+PLT+ in each monocyte subset from 16 independent experiments (**C**). Percentage of monocytes with activated bound platelets (PLT+CD62P+) in each CD14+PLT+ monocyte subset from 16 independent experiments. Statistical analysis was performed using one-way ANOVA with Tukey’s multiple comparisons test. *** *p* < 0.001.

We observed higher percentages of PLT+ and PLT+CD62P+ in CD14+CD16+ and CD14loCD16++ than in CD14+CD16- (Figure 1A–C).

When we compared CD14+PLT+ and CD14+PLT-, we did not find differences in the expression of chemokine receptors (CD15, CCR2), complement receptor (CD35) and certain adhesion molecules (CD31, CD49d, CD52, CD62L). However, CD14+PLT+ had a higher expression of antigen presentation molecules (HLA-DR, CD86), adhesion molecule CD54 and Fc receptors (CD16, CD64) than CD14+PLT- (HLA-DR: 6.12 ± 2.28, *p* < 0.001; CD86: 3.39 ± 1.04, *p* = 0.007; CD54: 2.33 ± 1.36, *p* = 0.008; CD16: 2.59 ± 1.53, *p* < 0.001; CD64: 1.22 ± 0.57, *p* < 0.001). CD14+PLT+CD62P+ and CD14+PLT+CD62P- monocytes had a higher expression of CD86, CD54, CD16 and CD64 than CD14 + PLT-. CD14+PLT+CD62P+ monocytes had a more elevated expression of HLA-DR and CD54 than CD14+PLT+CD62P- (Table S1, Figure S1).

**Figure 2:**
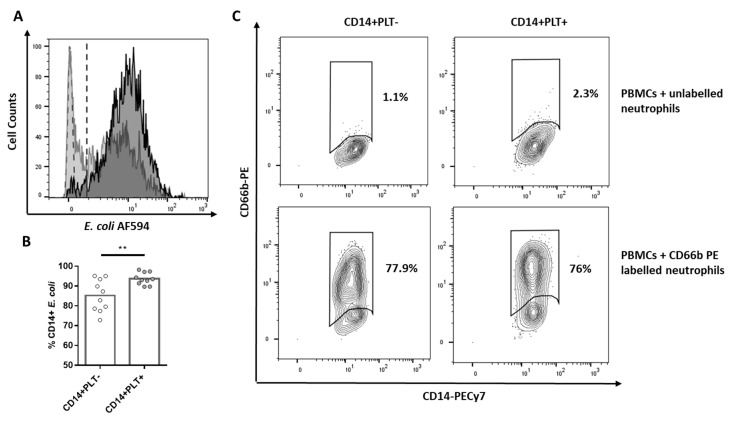
*E. coli* phagocytosis of HD monocytes with or without bound platelets. PBMCs were cultured with *E. coli* bioparticles stained with Alexa Fluor 594. (**A**) The representative overlapping histogram plot is shown with the phagocytosis of CD14+PLT- in light gray and of CD14+PLT+ in dark gray. PBMCs without *E. coli* (light green dotted histogram) are the control to set up threshold of negative cells. Dotted line indicates positivity threshold. (**B**) Phagocytosis of *E. coli* by CD14+PLT- and CD14+PLT+ from 10 independent experiments. (**C**) Phagocytosis of apoptotic neutrophils (efferocytosis). A representative experiment is shown (*n* = 8) from 6 independent experiments. Data are expressed as percentages of gated monocytes. The statistical analysis was performed using the paired *t*-test. ** *p* < 0.01.

When we analyzed the phagocytic function, we found that CD14+PLT+ showed a greater capacity for the phagocytosis of *E. coli* bioparticles than CD14+PLT- (Figure 2A,B). However, we did not observe differences in efferocytosis (the phagocytosis of apoptotic neutrophils) between CD14+PLT+ and CD14+PLT- (Figure 2C).

**Figure 3:**
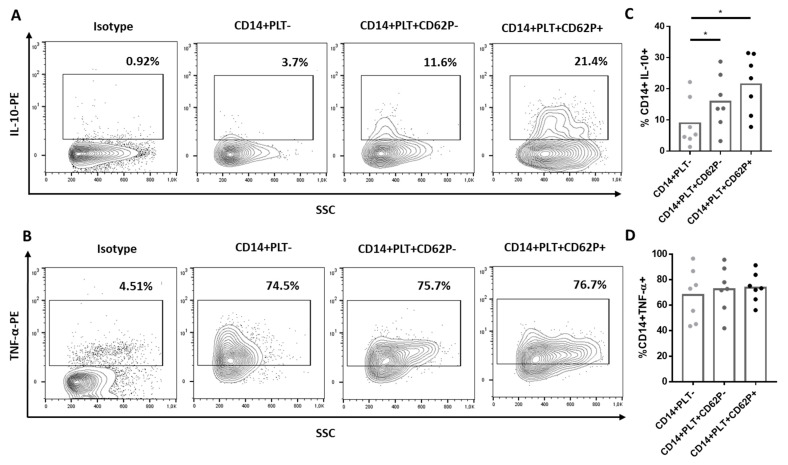
Cytokine production by monocytes without platelets (CD14+PLT-) and with non-activated (CD14+PLT+CD62P-) or activated (CD14+PLT+CD62P+) bound platelets. PBMCs were stimulated with LPS for 4 hours, and the secretion of IL-10 and TNF-α was analyzed by flow cytometry. A representative experiment is shown here (*n* = 7). The percentage of (**A**) IL-10+ or (**B**) TNF-α+ cells on CD14+PLT-, CD14+PLT+CD62P- or CD14+PLT+CD62P+ is shown. A comparison between percentages of (**C**) IL-10+ or (**D**) TNF-α+ cells on CD14+PLT-, CD14+PLT+CD62P- and CD14+PLT+CD62P+ is shown from 7 independent experiments. Statistical analysis was performed using one-way ANOVA with Tukey’s multiple comparisons test. * *p* < 0.05.

After culturing monocytes with LPS, we found that CD14+PLT+CD62P+ and CD14+PLT+CD62P- secreted more IL-10 than CD14+PLT- (Figure 3A,C). However, there were no differences in the secretion of TNF-α between CD14+PLT+CD62P+, CD14+PLT+CD62P- and CD14+PLT- monocytes (Figure 3B,D).

### 2.2. Comparison of Phenotype and Function of Monocytes with Bound PLTs in HD and SLE Patients

**Figure 4:**
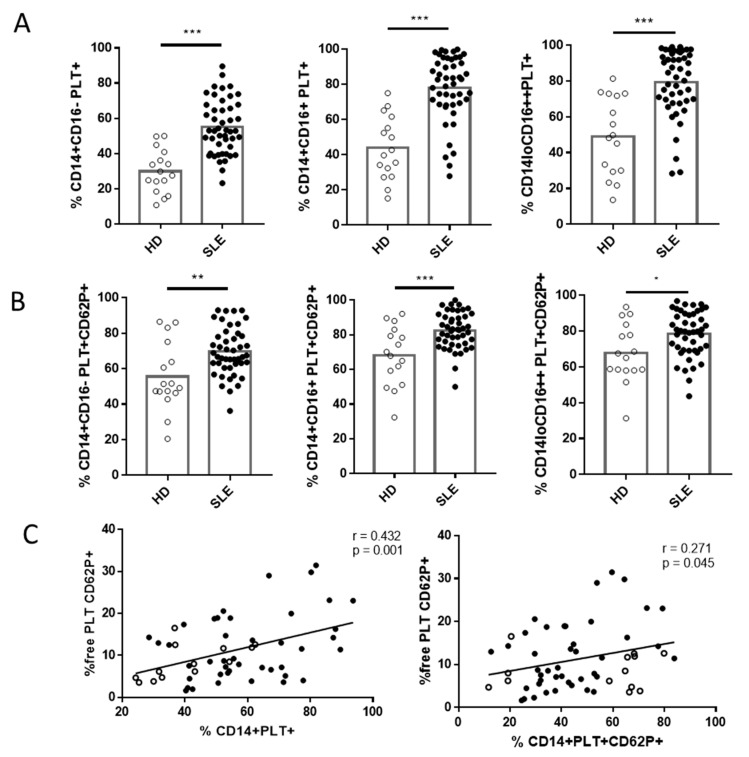
Monocytes with bound platelets and their relationship with free platelets in HD and SLE patients. (**A**) Percentage of monocyte subsets with bound platelets (PLT+) in HD and SLE patients. (**B**) Percentage of monocyte subsets with activated bound platelets (PLT+CD62P+) in HD and SLE patients. (**C**) Correlation between the percentage of CD14+PLT+ and CD14+PLT+CD62P+ with the percentage of free activated platelets (free PLT CD62P+) in HD and SLE patients. White circles represent HD (*n* = 16), and black circles represent SLE patients (*n* = 49). Statistical analysis was performed using the unpaired *t*-test (CD14+PLT+, CD14+PLT+CD62P+) for comparisons between HD and SLE and Spearman’s correlation for correlation analysis. * *p* < 0.05, ** *p* < 0.01, and *** *p* <0.001.

In line with recent reports [21], we found that patients with SLE had a lower percentage of CD14+CD16- (88.49 ± 5.9 for SLE vs. 92.72 ± 2.59 for HD, *p* = 0.017) and a higher percentage of CD14loCD16++ (6.21 ± 3.1 for SLE vs. 3.74 ± 1.19 for HD, *p* = 0.017) than HD. Interestingly, each subset of SLE monocytes had a higher percentage of monocytes with bound PLTs (Figure 4A), and the percentages of classical and intermediate monocytes with activated bound PLTs were also higher in SLE patients than in HD (Figure 4B). When we compared absolute counts, there were fewer monocytes (459.2 ± 176.8 monocytes/µL for SLE vs. 742.6 ± 94.5 monocytes/µL for HD, *p* < 0.001) and fewer monocytes without PLTs (201 ± 49.8 CD14+PLT-/µL for SLE vs. 425.9 ± 53.29 CD14+PLT-/µL for HD, *p* < 0.001) in SLE patients than in HD, but comparable levels of CD14+PLT+ counts (data not shown). No correlation was found between the percentages of CD14+PLT+ and PLT counts (r = 0.130, *p* = 0.412). However, we observed a significant correlation between the percentage of CD14+PLT+ and CD14+PLT+CD62P+ (r = 0.940, *p* < 0 001 for SLE; r = 0.856, *p* = 0.006 for HD) and between the percentages of CD14+PLT+ and free PLT CD62P+ in SLE patients and HD (Figure 4C). We also found a tendency towards a higher percentage of free PLT CD62P+ in SLE patients than in HD (12.04 (6.73–18.93) for SLE vs. 8.01 (4.75–12.24) for HD, *p* = 0.06).

As in HD, a higher expression of HLA-DR, CD86, CD54, CD16 and CD64 was found on CD14+PLT+CD62P+ and CD14+PLT+CD62P- than on CD14+PLT- (Table S1). There were no differences in HLA-DR, CD54 and CD16 expression on monocytes between SLE patients and HD (data not shown). However, CD64 expression was higher on CD14+PLT- from SLE patients than from HD (1.18 ± 0.49 for SLE vs. 0.88 ± 0.29 for HD, *p* = 0.021), and CD86 expression was lower on CD14+PLT+CD62P+ from SLE patients than from HD (2.9 ± 0.9 for SLE vs. 3.87 ± 1.36 for HD, *p* = 0.023).

Although there were no differences in *E. coli* phagocytosis between monocytes from SLE patients and HD, we found, similarly to HD, a higher percentage of CD14+PLT+ than CD14+PLT- phagocytosed *E. coli* in SLE (Figure S2A). On the other hand, monocytes from SLE patients showed less efferocytosis than HD monocytes but without differences between CD14+PLT+ and CD14+PLT- (Figure S2B).

Higher levels of plasma IFN-α, but not of IL-6 and IL-10, were observed in SLE patients than in HD (54.35 ± 23.04 for SLE vs. 7.3 ± 3.8 for HD, *p* = 0.029). No correlation between IFN-α, IL-6 or IL-10 with the percentage of CD14+PLT+ in SLE patients or in HD was observed (data not shown). Although there were no differences in the percentage of IL-10+ monocytes from SLE patients and HD, we found higher percentages of IL-10+ in CD14+PLT+CD62P- and CD14+PLT+CD62P+ than in CD14+PLT- monocytes (Figure S2C).

### 2.3. Correlation of PSGL-1 and CD40 and Their Ligands sCD62P and sCD40L with CD14+PLT+ in SLE Patients and HD

**Figure 5:**
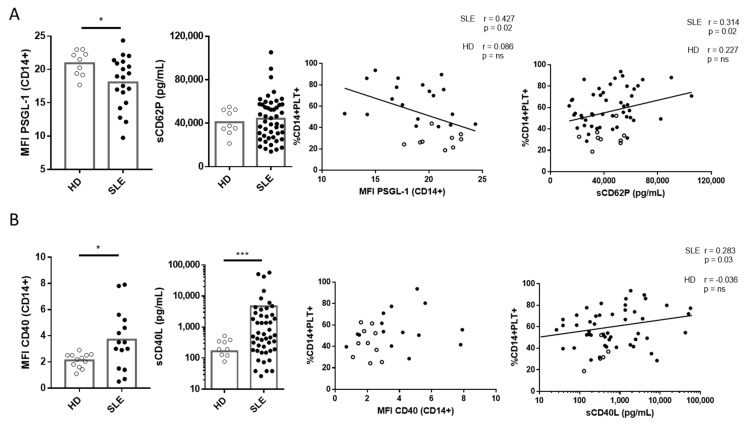
Expression of PSGL-1 and CD40 on monocytes and quantification of soluble CD62P (sCD62P) and CD40L (sCD40L) in plasma from HD and SLE patients and their association with the percentage of monocytes with bound platelets (CD14+PLT+). (**A**) PSGL-1 gMFI of monocytes from HD (*n* = 9) and SLE patients (*n* = 20). Levels of sCD62P in plasma from HD (*n* = 9) and SLE patients (*n* = 49). PSGL-1 and sCD62P correlations with the percentage of CD14+PLT+. (**B**) CD40 MFI of monocytes from HD (*n* = 12) and SLE patients (*n* = 15). Levels of sCD40L in plasma from HD (*n* = 9) and SLE patients (*n* = 49). CD40 and sCD40L correlations with the percentage of CD14+PLT+. White circles represent HD, and black circles represent SLE patients. Statistical analysis was performed using the Mann–Whitney test for comparisons between HD and SLE and Pearson’s (PSGL-1 and sCD62P) or Spearman’s (CD40 and sCD40L) correlation for correlation analysis. * *p* < 0.05, *** *p* < 0.001.

We observed lower levels of PSGL-1 expression on monocytes from SLE than HD but no differences in the plasmatic sCD62P levels of SLE patients and HD. Differences in PSGL-1 expression between CD14+PLT-, CD14+PLT+CD62P- and CD14+PLT+CD62P+ were not observed in HD nor in SLE patients (data not shown). The percentage of CD14+PLT+ in SLE, but not in HD, correlated inversely with the expression levels of PSGL-1 and directly with plasma sCD62P levels (Figure 5A). The percentage of CD14+PLT+CD62P+ did not correlate with the expression of PSGL-1 on monocytes or plasma sCD62P in SLE patients or HD (data not shown).

We found higher levels of CD40 on monocytes from SLE patients than HD and higher plasmatic sCD40L levels in SLE patients than in HD. No correlation was found between CD40 expression on monocytes and the percentage of CD14+PLT+ or CD14+PLT+CD62P+ in SLE patients or HD (Figure 5B and data not shown). However, we observed that plasmatic sCD40L levels correlated with CD14+PLT+ but not with CD14+PLT+CD62P+ in SLE patients (Figure 5B and data not shown).

### 2.4. Relationship between CD14+PLT+ and Clinical Features in SLE Patients

**Figure 6:**
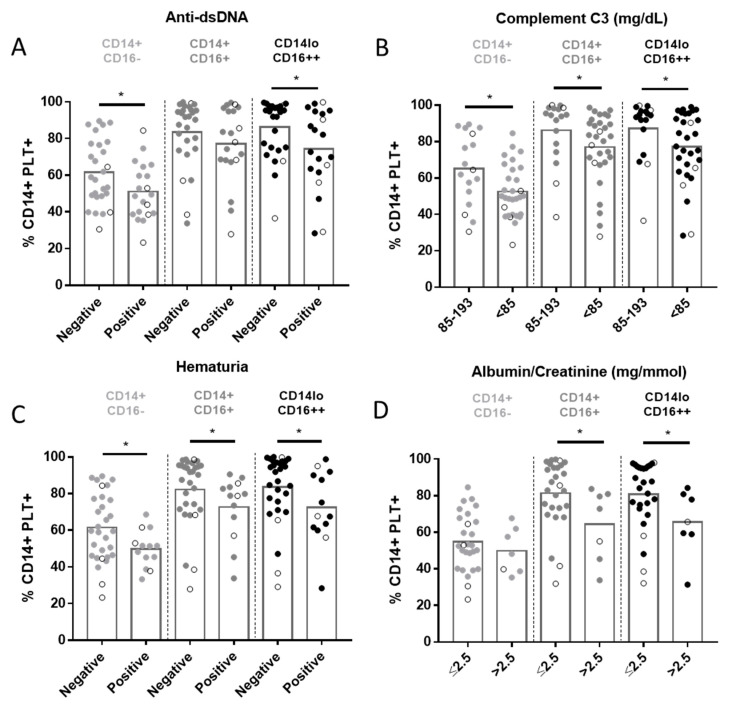
Percentage of monocyte subsets with bound platelets in SLE patients and clinical features. Comparisons of the percentages of monocyte subsets with bound platelets (CD14+PLT+) in SLE patients segregated according to (**A**) positive or negative anti-dsDNA, (**B**) <85 or 85–193 mg/dL of C3, (**C**) positive or negative hematuria and (**D**) >2.5 or ≤2.5 mg/mmol of albumin/creatinine are shown (*n* = 49). Patients undergoing treatment with mycophenolate are marked with white circles. The statistical analysis was performed using the unpaired *t*-test. * *p* < 0.05.

We did not find any association between CD14+PLT+ and SLEDAI score (r = 0.19; *p* = 0.18). However, as SLEDAI is an activity index that integrates different parameters, we then analyzed them independently. SLE patients with anti-dsDNA antibodies had a lower percentage of CD14+CD16-PLT+ and CD14loCD16++PLT+ than those with undetectable levels of anti-dsDNA antibodies (Figure 6A). A negative correlation was found between the levels of anti-dsDNA antibodies and the percentage of CD14+CD16+PLT+, CD14loCD16++PLT+, CD14+CD16+PLT+CD62P+ and CD14loCD16++PLT+CD62P+ (Figure S4). SLE patients with anti-Sm antibodies had a lower percentage of the three monocyte subsets with bound PLTs (Figure S3A). SLE patients with anti-C1q antibodies also had a lower percentage of CD14+CD16+ and CD14loCD16++ with bound PLTs (Figure S3B). There were no differences in the percentage of CD14+PLT+ when patients were segregated according to the presence of anti-SSA, anti-SSB, anti-U1RNP or anti-phospholipid antibodies (data not shown). There were no differences either in the percentage of CD14+PLT+CD62P+ when segregated according to anti-dsDNA (Figure S3C), anti-Sm, anti-C1q, anti-SSA, anti-SSB, anti-U1RNP or anti-phospholipid antibodies (data not shown).

SLE patients with low C3 levels (<85 mg/dL) and those with hematuria had lower percentages of the three subsets of CD14+PLT+ than patients with normal C3 levels (85–193 mg/dL) and those without hematuria (Figure 6B,C), respectively. There were no differences in the percentage of CD14+PLT+CD62P+ when segregating according to C3 levels (Figure S3E). SLE patients with hematuria had lower percentages of CD14+CD16+PLT+CD62P+ than SLE patients without hematuria (Figure S3D).

SLE patients with albumin/creatinine levels >2.5 mg/mmol showed lower percentages of CD14+CD16+PLT+, CD14loCD16++PLT+ and CD14+CD16+PLT+CD62P+ than those with normal levels (≤2.5 mg/mmol) (Figure 6D and Figure S3F). Comparable percentages of each subset of CD14+PLT+ were observed when SLE patients were segregated according to cutaneous or articular manifestations or according to prednisone, hydroxychloroquine and azathioprine medication. Interestingly, lower percentages of CD14+PLT+ were observed when SLE patients were segregated according to treatment with or without mycophenolate (48.08 ± 15.32 treated patients; 62.31 ± 15.5 not-treated patients; *p* = 0.028).

## 3. Discussion

Our results showed that PLTs can bind to monocytes, altering their phenotype and function. HD and SLE monocytes with bound PLTs and, especially, with activated bound PLTs showed an up-regulation of co-stimulation, adhesion and Fc receptor molecules, and an increased phagocytosis and IL-10 secretion. However, the percentages of monocytes with bound PLTs and with activated bound PLTs were higher in SLE than in HD. In addition, we found that monocytes from HD and SLE had a different expression of the key molecules responsible for PLT binding to monocytes, suggesting that each binding generates a characteristic signaling. The association of monocytes with PLTs with SLE clinical characteristics suggests that the increased binding and different signaling of PLTs to monocytes may be factors contributing to SLE pathology.

We showed that CD14+PLT+, and especially CD14+PLT+CD62P+, had an increased expression of HLA-DR, CD86, CD54, CD16 and CD64, suggesting, in line with other studies, an increased activation state [14,46], antigen presentation, adhesion capacity and phagocytosis of opsonized particles [47,48]. These findings are in concordance with other authors that previously reported an up-regulation of CD86 and CD16 on monocytes with bound PLTs compared with those without bound PLTs in HD [16,18]. However, with current experiments we are not able to conclude whether the binding of PLTs to monocytes induces the up-regulation of these markers or whether PLTs bind to monocytes with an activated state.

Although we found that CD14+PLT+ had an increased phagocytosis of *E. coli*, monocytes with bound PLTs did not have more efferocytosis ability than CD14+PLT-. It has been described that PLTs can recognize bacteria and their products via TLR4 and opsonize them, inducing subsequent phagocytosis [49,50,51,52]. Therefore, it is likely that the increased phagocytosis of CD14+PLT+ is due to an enhanced adhesion caused by the additional TLR4 expression from the PLTs. It remains to be determined whether the monocyte and/or the bound PLTs are responsible for *E. coli* binding. This explanation cannot be applied to efferocytosis because the “eat-me signals” responsible for efferocytosis are recognized by a series of membrane and soluble receptors that are not on PLTs.

Our results showed that CD14+PLT+, especially CD14+PLT+CD62P+, had an increased percentage of IL-10-secreting cells compared with CD14+PLT-. Previous studies have found that CD14+CD16+ monocytes are the main IL-10 producers [53]. Since we observed that CD14+CD16+ monocytes were the subset of monocytes with the most bound PLTs, this fact could explain the greater IL-10 production by CD14+PLT+. Previous studies have described an increase in IL-10 production by monocytes when PBMCs were co-cultured with PLT-rich plasma [18]. These authors found that soluble factors are the main molecules responsible for the increase in IL-10. However, in our experimental system, where there was no external supply of activated PLTs, we detected that both bound PLT+CD62P- and PLT+CD62P+ are associated with a higher IL-10 secretion by monocytes. Despite the IL-10 differences, we found comparable percentages of TNF-α-secreting cells between CD14+PLT+ and CD14+PLT-. Gudbrandsdottir et al. described a reduction in TNF-α secretion by PBMCs co-cultured with activated PLTs. They hypothesized that increased production of IL-10 by PBMCs down-regulates TNF-α secretion. It is possible that, in our experiments, without additional activated platelets, the amount of IL-10 was not enough to down-regulate TNF-α. Furthermore, Gudbrandsdottir et al. measured the released TNF-α in culture supernatants, and differences in methodology may also explain the discrepancies in results.

We found that the three monocyte subsets of SLE patients had an increased percentage of bound PLTs and activated PLTs compared with HD. In line with this, Joseph et al. described an increase in CD14+PLT+ and higher PLT activation in SLE patients [54]. Both our findings and those of Joseph et al. are consistent with previous observations arguing that the percentage of CD14+PLT+ is a marker of PLT activation [40].

Upon analyzing the molecules that may be involved in the binding of PLTs to monocytes, we found that SLE monocytes with and without platelets have a lower expression of PSGL-1 than HD. Some authors have reported the down-regulation of PSGL-1 on myeloid innate cells in an inflammatory context. Marsik et al. found that endotoxin down-regulates PSGL-1 expression on human monocytes [55]. Other authors have observed a down-regulation of PSGL-1 on monocytes from HIV patients or after leukocyte activation [56,57]. Further experiments will reveal whether the lower expression of PSGL-1 on monocytes in all these pathologies is due to a down-regulation or to an increased shedding. Interestingly, in SLE patients, this PSGL-1 expression is inversely correlated with the percentage of CD14+PLT+. In our experimental system we cannot rule out that the lower expression of PSGL-1 may be due to a misdetection due to PLT binding. However, this explanation is unlikely because in HD we did not find any correlation between PSGL-1 and the percentage of CD14+PLT+. Additionally, since we found a decreased detection of PSGL-1 on monocytes from SLE patients, we can speculate that other molecules could be contributing to monocyte–PLT interaction. Thus, we found a higher expression of CD40 on monocytes in SLE patients compared with HD. This finding, along with the lower PSGL-1 expression on SLE monocytes and the higher activation of PLTs (by the surrogate marker sCD40L), suggests that CD40-CD40L is another pair that contributes to the binding of PLTs to monocytes in SLE patients. In line with this, Gudbrandsdottir et al. found that activated PLTs had anti-inflammatory properties related to the interaction between CD40L and CD40 [18]. It has been hypothesized that an aberrant expression of CD40 on SLE monocytes may promote the proliferation of autoreactive lymphocytes and the generation of autoantibodies through excess CD40–CD40L interactions [58]. Therefore, it is tempting to speculate that the ligation of CD40 on monocytes with CD40L on PLTs prevents monocyte–lymphocyte interaction and the generation of autoantibodies.

Regarding PLT activation, we found a tendency towards a higher percentage of free PLT CD62P+ and higher percentages of monocytes with bound PLTs in SLE than in HD, which are two markers of PLT activation [40]. Although we did not find differences in sCD62P plasma levels between SLE and HD, Joseph et al. observed increased plasma sCD62P in SLE [8]. This apparent discrepancy may be due to differences between the two cohorts of patients, since the patients in Joseph et al. were younger than those in our cohort, and PLT activation status is thought to be affected by age [59]. In line with PLT activation, we also observed an increased concentration of plasma sCD40L in patients with SLE than in HD. Although lymphocytes also secrete sCD40L [60], sCD40L increase is more likely due to PLT activation. Some authors have reported an increased activation of PLTs from SLE patients [61].

The lowest percentages of CD14+PLT+ were found in SLE patients with active disease and renal manifestations. This seems to contradict our previous studies, which showed that SLE patients with active disease and renal manifestations had the highest percentages of B and T lymphocytes with bound PLTs [62]. However, we found no correlation between the percentages of myeloid and lymphoid cells with bound PLTs (Figure S5) in these patients. There are at least two possible explanations for this discrepancy. First, monocytes are more sensitive to PLT activation than lymphocytes [63]. Second, numerous pairs of molecules have been identified as participating in the binding of PLTs to monocytes, while only a few have been identified in the binding of PLTs to lymphocytes (CD62P-PSGL-1, GPIb-CD11b, CD40-CD40L, GPIIb/IIIa-CD11/CD18) [64]. We can therefore speculate that there is a different functional consequence of PLT binding to myeloid or lymphoid cells when caused by a different signaling molecule.

Although we found an association of monocytes with bound PLTs with more IL-10 production but with less autoantibodies, we did not find an association of IL-10 with autoantibodies or disease activity. Concordantly, other studies have not shown IL-10 association with disease activity [65]. Furthermore, treatment with anti-IL-10 antibody causes joint and cutaneous improvement, but not anti-dsDNA decrease [66], and anti-IL-10 autoantibodies are related to increased serum IgG levels [67]. However, others have described that IL-10 acts as a growth, differentiation [68] and anti-apoptotic factor on B lymphocytes [69] and that this cytokine correlates with disease activity and dsDNA titers [70]. The apparent contradiction between IL-10 and autoantibodies in different reports of SLE could be explained by different treatments, disease activity or demographic characteristics as well as different quantification methods. Further studies taking into account all these factors should be performed to establish a definitive relationship between IL-10 and autoantibodies in SLE.

Our study has some limitations. One is that we did not analyze the influence of monocytes with bound PLTs over other leukocytes. We could speculate that the binding of PLTs to monocytes could regulate membrane and soluble molecules involved in neutrophil attraction and stimulation and/or lymphocyte activation. Another limitation of our study is that we excluded patients receiving a dose of glucocorticoids higher than 10 mg/day in order to avoid the influence of this treatment on results. This exclusion may have biased the cohort to a less severe one, and it may explain the low frequency of patients with anti-dsDNA antibodies compared with other cohorts [71]. With our current approach, we cannot discriminate whether the increased percentage of CD14+PLT+ is involved in the development of SLE or if it is a consequence of the disease status. Further studies analyzing the same patient in terms of flare and remission will reveal whether monocyte-PLT complexes are a useful tool for following up SLE activity and clinical manifestations. Nevertheless, our findings suggest that a better understanding of monocyte–PLT interaction would be beneficial for the therapeutic regulation of autoimmunity.

## 4. Materials and Methods

### 4.1. Study Subjects and Sample Collection

Whole blood from 16 HD and 49 SLE patients was collected in heparin-BD vacutainer tubes (BD, Franklin Lakes, NJ, USA). SLE diagnosis was based on 1982 revised ACR criteria [72]. To assess disease activity, Systemic Lupus Erythematous Disease Activity Index (SLEDAI) was calculated at the time of sample collection [73]. The demographic, clinical and laboratory data of SLE patients enrolled in this study are shown in Table 1. Written informed consent was obtained and ethical approval for the study was granted by the Hospital de la Santa Creu I Sant Pau Institutional Ethics Committee.

### 4.2. Staining of Whole Blood Cells and Flow Cytometry Analysis

Whole blood (100 μL) was incubated with anti-CD16 AF647 clone 3G8, anti-CCR2 AF647 clone K036C2 (BioLegend, San Diego, CA, USA), anti-CD15 PE clone MEM-158, anti-CD31 PE clone MEM-05, anti-CD35 FITC clone UT11, anti-CD41a-FITC, anti-CD41a-APC clone HIP8, anti-CD52 PE clone HI186, anti-CD62P-PE, anti-CD62P-APC clone HI62P, anti-HLA-DR-FITC clone MEM-12, anti-CD54-PE clone 1H4 (Immunotools, Friesoythe, Germany), anti-CD14-PECy7 clone M5E2, anti-CD49d FITC clone 9F10, anti-CD62L APC clone DREG-56, anti-CD64-PE clone 10.1 and anti-CD86-PE clone 2331 (FUN-1) (BD Biosciences, San Jose, CA, USA). Red blood cells were lysed and white cells were fixed using BD FACS lysing solution (BD Biosciences) to be analyzed by flow cytometry. Samples were acquired with the MACS-Quant Analyzer 10 flow cytometer (Miltenyi Biotec, Bergisch Gladbach, Germany), and we determined the count of cells/µL, the percentages of cells and the geometric mean fluorescence intensity (gMFI) using FlowJo vX. CD41a+ PLTs/µL were quantified by flow cytometry. Activated PLTs were identified as CD41a+CD62P+. Monocytes were gated according to the expression of CD14. Three subsets of monocytes were gated according to the expression of CD14 and CD16 [74]: classical (CD14+CD16-), intermediate (CD14+CD16+) and non-classical monocytes (CD14loCD16++). Monocytes with bound PLTs were identified as CD41a+, and those with bound activated PLTs were identified as CD41a+CD62P+.

### 4.3. IL-10 and TNF-α Secretion Assays

IL-10 or TNF-α production was analyzed on CD14 +PLT-, CD14 +PLT+CD62P-, and CD14+PLT+CD62P+ monocytes after ultrapure TLR4 agonist lipopolysaccharide (LPS) (Invivogen, San Diego, CA, USA) stimulation using the IL-10 or TNF-α secretion assay (MiltenyiBiotec), as previously described [62]. Briefly, after 4 hours of LPS stimulation (1 µg/mL), PBMCs were labeled with IL-10- or TNF-α-specific catch reagent and incubated under slow rotation. Cells were washed and labeled with the IL-10-PE or TNF-α-PE detection antibody, CD14-PECy7 (BD Biosciences), CD41a-FITC and CD62P-APC (Immunotools), and the viability marker LIVE/DEAD fixable Violet Dead Cell Stain kit (Thermo Fisher Scientific, Waltham, MA, USA). Membrane IL-10 or TNF-α was analyzed by flow cytometry.

### 4.4. Phagocytosis Assays

PBMCs were incubated with *E. coli* bioparticles conjugated with Alexa Fluor 488 (Invitrogen, Karlsruhe, Germany) at a proportion of 1:5 for 2 hours at 37 °C and at 4 °C. Cells were then surface stained with anti-CD14-PECy7 and anti-CD41a-APC and analyzed by flow cytometry. We included two negative controls. One was samples incubated at 4 °C, a condition to abolish metabolism for discarding surface binding. The second negative control was samples incubated at 37 °C without *E. coli* Alexa Fluor 488, to establish cell autofluorescence. Then, *E. coli* phagocytosis was established as the percentage of CD14+ cells that were positive for Alexa Fluor 488 at 37 °C.

### 4.5. Apoptosis Analysis of Neutrophils and Efferocytosis Assays

Neutrophils were collected from the pellet after Ficoll centrifugation (Ficoll-Hypaque gradient centrifugation). Red blood cells were removed by RBC Lysis Buffer (Biolegend). Neutrophils (1 × 106 cells/mL) were left for 48 hours in 5% CO_2_ at 37 °C in complete medium. Apoptotic and necrotic neutrophil rates were analyzed by flow cytometry after staining with anti-CD66b-PE (BD Biosciences), FITC-conjugated Annexin V (Immunotools) and Propidium-Iodide (Bender medsystems, ebioscience, San Diego, CA, USA), as previously described [75]. PBMCs were co-cultured for four hours at 37 °C at a 1:4 ratio with apoptotic neutrophils previously stained with anti-CD66b-PE or unstained for fluorescence minus one (FMO) control. To discard surface binding, the co-culture of neutrophils and PBMCs was also performed at 4 °C. After co-culture, cells were washed twice with PBS and surface stained with anti-CD14-PECy7, anti-CD41a-FITC and anti-CD62P-APC. We did not observe CD14+CD66b+ cells in samples incubated at 4 °C. Monocytes that had engulfed apoptotic neutrophils were identified as CD14+CD66b+ cells by flow cytometry according to FMO control.

### 4.6. Determination of IFN-α, IL-6, IL-10, sCD62P and sCD40L Levels

Plasma concentrations of IFN-α (Mabtech, Stockholm, Switzerland), IL-6 (Immunotools), IL-10 (Immunotools), sCD62P (R&D Systems, Minneapolis, MN, USA) and sCD40L (Peprotech, London, UK) were determined using specific ELISA kits according to the manufacturers’ instructions and using the specific standard curves of recombinant molecules. The limits of detection were as follows: 2 pg/mL for IFN-α, 8 pg/mL for IL-6, 16 pg/mL for IL-10, 125 pg/mL for sCD62P and 31.25 pg/mL for sCD40L.

### 4.7. Quantification of Autoantibodies, C3, Albumin/Creatinine and Hematuria

Anti-dsDNA, Sm, SSB-La, SSA-Ro and U1-RNP levels were determined in serum using BIO-FLASH® chemiluminescence (Werfen, Barcelona, Spain). Anti-C1q levels in serum were quantified by Quanta Lite Anti-C1q ELISA (Inova Diagnostic, San Diego, CA, USA). Anti-histone presence in serum was established using Euroline ANA Perfil (Euroimmune, Lübeck, Germany). Levels of C3 in serum were determined using the Nephelometry System (Beckman Coulter Diagnostics, Nyon, Switzerland). Albumin/creatinine ratio was determined in serum by the turbidimetry system (Abbott, Chicago, IL, USA). The presence of hematuria was determined by the Combur test (Roche, Basilea, Switzerland). Anti-dsDNA antibodies were considered positive when levels were >35 UI/mL. Sm, SSB-La, SSA-Ro and U1-RNP antibodies were considered positive when levels were >20 Chemoluminiscence Units and C1q when levels were >20 U/mL according to manufacturer’s study. C3 levels were considered decreased when they were below 85 mg/dL according to manufacturer’s study. Microalbuminuria was considered when albumin/creatinine ratio was >2.5 mg/mmol.

### 4.8. Statistics

Statistical analyses were performed using Graph PadPrism 7 software. The Kolmogorov–Smirnov test was applied to test the data for normal distribution. Normally distributed variables were then reported as mean ± SD, and non-parametric distributed variables were reported as median (interquartile range) (IQR). SLEDAI was expressed as mean (range). Comparisons of 3 groups were tested with one-way analysis of variance (ANOVA) and Tukey’s post-hoc test. Comparisons between 2 groups were tested with the student’s (paired or unpaired) *t*-test, Mann–Whitney test, or Wilcoxon test according to Gaussian distribution. Correlation analyses were carried out with Pearson’s or Spearman’s correlation according to Gaussian distribution. *P*-values < 0.05 were considered significant.

## Figures and Tables

**Table 1:** Demographic, clinical and laboratory characteristic data of study patients.

General Conditions	SLE Patients	Healthy Donors
Age in years, mean ± SD	50.4 ± 15.29	52.2 ± 17.7
Gender, % (*n*) women	93.75 (45)	90 (18)
Years of development, median (IQR)	9 (3–17)	
SLEDAI, mean (range)	2.89 (0–10)	
**Laboratory parameters**		
C3 (mg/dL, median (IQR))	75.1 (63.03–100.8)	
Decreased C3 or C4, % (*n*)	66.7 (32)	
Positive anti-dsDNA, % (*n*)	41.7 (20)	
Positive ANA, % (*n*)	91.7 (44)	
Positive anti-Sm, % (*n*)	23 (11)	
Positive anti-C1q, % (*n*)	43 (20)	
Positive anti-SSB-La, % (*n*)	16 (7)	
Positive anti-SSA-Ro, % (*n*)	44 (20)	
Positive anti-U1-RNP, % (*n*)	30 (14)	
Positive antiphospholipid antibody, % (*n*)	27 (13)	
Leukopenia and/or lymphopenia, % (*n*)	37.5 (18)	
**Clinical features, %** (*n*)		
Renal involvement	41.7 (20)	
Hematuria	27.1 (13)	
Albumin/creatinine > 2.5	14.6 (7)	
Cutaneous involvement	18.8 (9)	
Arthritis	14.6 (7)	
**Treatment, %** (*n*)		
None	6 (3)	
Mycophenolate	17 (8)	
Prednisone	37 (18)	
Hydroxychloroquine	79 (38)	
Azathioprine	8 (4)	

## Data Availability

The data presented in this study are available on request from the corresponding author. The data are not publicly available due to ethical restriction.

## Supplementary Materials

The following are available online at https://www.mdpi.com/article/10.3390/ijms22094719/s1, Figure S1. Phenotype of monocytes without platelets (CD14+PLT-) and with non-activated (CD14+PLT+CD62P-) or activated (CD14+PLT+CD62P+) platelets in HD. Figure S2. Functional characterization of monocytes with bound platelets in SLE patients. Figure S3. Percentage of monocyte subsets with bound platelets and activated bound platelets in SLE patients according to clinical features. Figure S4. Correlation of monocyte subsets with bound platelets and activated bound platelets with anti-dsDNA titers in SLE patients. Figure S5. Correlation of monocytes with bound platelets and lymphocytes with bound platelets in SLE patients. Table S1. Expression of HLA-DR, CD86, CD54, CD16 and CD64 on monocytes without bound platelets (CD14+PLT-) and monocytes with non-activated (CD14+PLT+CD62P-) or activated bound platelets (CD14+PLT+CD62P+) in HD and SLE patients.
