# Supplementary material for: Phenotypic and Functional Consequences of PLT Binding to Monocytes and Its Association with Clinical Features in SLE
Source: Int J Mol Sci. 2021 Apr 29;22(9):4719. doi: 10.3390/ijms22094719 (PMC8125177; doi:10.3390/ijms22094719)
Supplement: Supplementary file 1 [file ijms-22-04719-s001.zip › ijms-1169242-supplementary/ijms-1169242-supplementary.pdf]

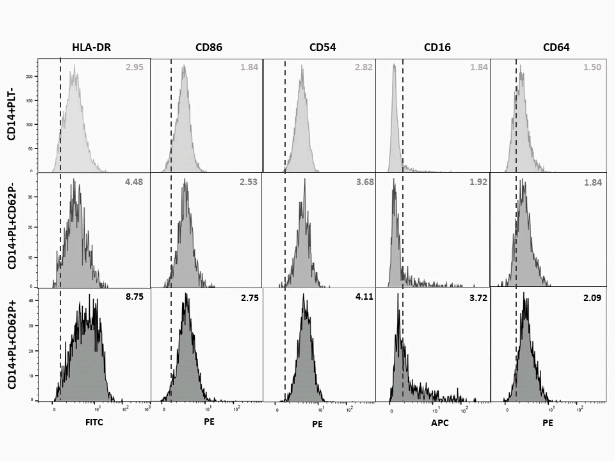

Figure S1. Phenotype of monocytes without platelets (CD14+PLT-) and with non-activated (CD14+PLT+CD62P-) or activated (CD14+PLT+CD62P+) platelets in HD. Expression of HLA-DR, CD86, CD54, CD16 and CD64 on CD14+PLT- (light grey), CD14+PLT+CD62P- (dark grey) or CD14+PLT+CD62P+ (black). Dotted line indicates positivity threshold. Representative experiment is shown from 16 independent experiments. Data are expressed as gMFI (geometric mean fluorescence intensity) of gated monocytes.

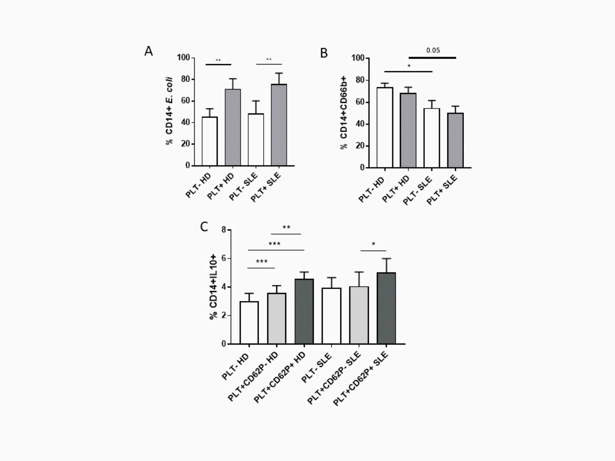

Figure S2. Functional characterization of monocytes with bound platelets in SLE patients. (A) Phagocytosis of *E. coli* (n=8), (B) Efferocytosis (n=8) and (C) IL-10 secretion (n=9). Error bars correspond to SEM. Data are shown for 3 (A), 8 (B) and 6 (C) independent experiments. Statistical analysis was performed using the paired t-test for comparison between monocytes from HD or from SLE patients and t-test or the one-way ANOVA with Tukey's multiple comparisons test for comparison between HD and SLE patients. ∗p<0 05, ∗∗p<0 01, and ∗∗∗p<0 001.

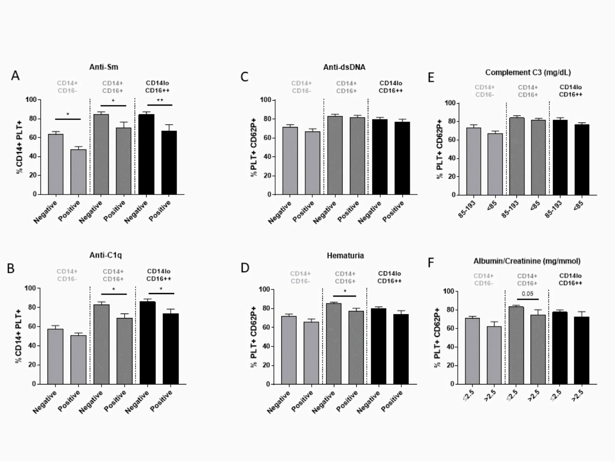

Figure S3. Percentage of monocytes subsets with bound platelets and activated bound platelets in SLE patients according to clinical features. Comparison of percentages of monocyte subsets with bound platelets (CD14+PLT+) in SLE patients segregated according to (A) positive or negative anti-Sm, (B) positive or negative anti-C1q. Comparison of percentages of monocyte subsets with activated bound platelets (PLT+ CD62P+) in SLE patients segregated according to (C) positive or negative anti-dsDNA, (D) positive or negative haematuria, (E) <85 or 85-193 mg/dl of C3 and (F) >2.5 or ≤2.5 mg/mmol of albumin/creatinine are shown (n=49). Error bars correspond to SEM. Statistical Statistical analysis was performed using the t-test. ∗p <0.05, **p<0.01.

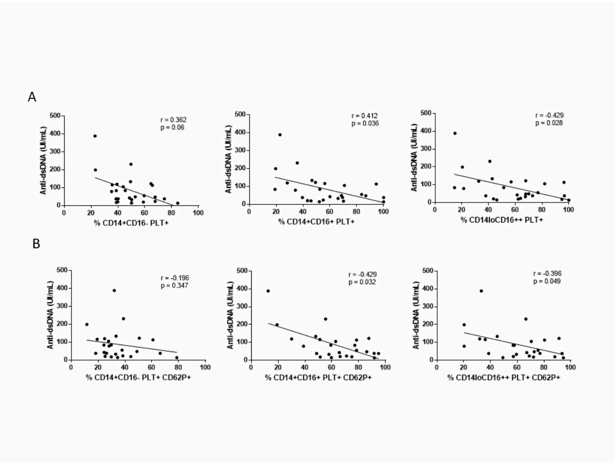

Figure S4. Correlation of monocyte subsets with bound platelets and activated bound platelets with anti-dsDNA titers in SLE patients. (A) Correlation of the percentage of monocyte subsets with bound platelets (PLT+) with anti-dsDNA titers in SLE patients (n=26). (B) Correlation of percentage of monocyte subsets with activated bound platelets (PLT+ CD62P+) with anti-dsDNA titers in SLE patients (n=26). Statistical analysis was performed using Pearson's correlation.

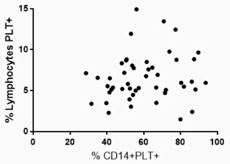

Figure S5. Correlation of monocyte with bound platelets and lymphocytes with bound platelets in SLE patients. (n=48) Statistical analysis was performed using Pearson's correlation.

Table S1. Expression of HLA-DR, CD86, CD54, CD16 and CD64 on monocytes without bound platelets (CD14+PLT-), monocytes with non-activated (CD14+PLT+CD62P-) or activated bound platelets (CD14+PLT+CD62P+) in HD and SLE patients. Data are expressed as the mean ± SD values of gMFI of 35 independent experiments (16 HD and 35 SLE patients). Statistical analysis was performed using the one-way ANOVA with Tukey's multiple comparisons test. gMFI, geometric mean fluorescence intensity, SD, standard desviation.

		CD14+PLT- (gMFI, x̄ ± SD) (a)	CD14+PLT+CD62P- (gMFI, x̄ ± SD) (b)	CD14+PLT+CD62P+ (gMFI, x̄ ± SD) (c)	ANOVA	(a) Vs. (b)	(a) Vs. (c)	(b) Vs. (c)
HLA-DR	HD	4.27 ± 1.34	4.64 ± 1.66	7.67 ± 2.8	**<0.01**	ns	**<0.01**	**<0.01**
	SLE	4.18 ± 1.25	4.37 ± 1.5	7.63 ± 2.4	**<0.01**	ns	**<0.01**	**<0.01**
CD86	HD	2.43 ± 0.58	2.87 ± 0.56	3.87 ± 1.36	0.07	**<0.01**	**<0.01**	ns
	SLE	2.14 ± 0.65	2.41 ± 0.65	2.9 ± 0.9	**<0.01**	**<0.01**	**<0.01**	**<0.01**
CD54	HD	1.87 ± 0.5	2.25 ± 0.8	2.92 ± 1.66	**0.04**	**<0.01**	**<0.01**	ns
	SLE	2.06 ± 0.63	2.41 ± 0.69	2.83 ± 0.69	**<0.01**	**<0.01**	**<0.01**	**<0.01**
CD16	HD	1.7 ± 0.89	2.33 ± 1.25	2.8 ± 1.85	**0.01**	**0.04**	**0.02**	ns
	SLE	1.84 ± 1.08	2.48 ± 2.05	2.6 ± 1.55	**0.03**	ns	**<0.01**	ns
CD64	HD	0.88 ± 0.29	1.12 ± 0.51	1.18 ± 0.4	**0.01**	ns	**<0.01**	ns
	SLE	1.18 ± 0.49	1.26 ± 0.45	1.43 ± 0.49	**<0.01**	**0.01**	**<0.01**	**<0.01**
